# Evolution of the Influenza A Virus: Some New Advances

**Published:** 2008-01-30

**Authors:** Raul Rabadan, Harlan Robins

**Affiliations:** 1 Institute for Advanced Study, Einstein Dr., Princeton, NJ 08540, U.S.A.; 2 Fred Hutchinson Cancer Research Center, Seattle, Washington 98109, U.S.A.

**Keywords:** Influenza, human, Influenza, avian Influenza, seasonal Influenza, pandemic Influenza, antigenic drift, antigenic shift, reassortment, Spanish flu

## Abstract

Influenza is an RNA virus that causes mild to severe respiratory symptoms in humans and other hosts. Every year approximately half a million people around the world die from seasonal Influenza. But this number is substantially larger in the case of pandemics, with the most dramatic instance being the 1918 “Spanish flu” that killed more than 50 million people worldwide. In the last few years, thousands of Influenza genomic sequences have become publicly available, including the 1918 pandemic strain and many isolates from non-human hosts. Using these data and developing adequate bioinformatic and statistical tools, some of the major questions surrounding Influenza evolution are becoming tractable. Are the mutations and reassortments random? What are the patterns behind the virus’s evolution? What are the necessary and sufficient conditions for a virus adapted to one host to infect a different host? Why is Influenza seasonal? In this review, we summarize some of the recent progress in understanding the evolution of the virus.

The exponential increase in genomic sequence data over the last few years has led to improved understanding of three key features of Influenza evolution (Kamps, Hoffmann and Preiser, 2006; Lipatov, Govorkova, Webby, Ozaki, Peiris, Guan, Poon and Webster, 2004; Nelson and Holmes, 2007; Webster, Bean, Gorman, Chambers and Kawaoka, 1992). The first is Influenza’s capacity to target many different hosts. The main reservoirs of Influenza A are aquatic birds, mainly from the Orders Anseriformes (ducks, geese and swans) and Charadriiformes (gulls, terns and shorebirds). But, Influenza A viruses have been found in a large variety of other hosts, such as humans, pigs, horses, cats, dogs, seals, camels, whales. One of the causes of human Influenza pandemics is the jump, partially or completely, of a virus from a different host population into humans (Fitch, 1996; Gammelin, Altmuller, Reinhardt, Mandler, Harley, Hudson, Fitch and Scholtissek, 1990; Gorman, Bean, Kawaoka, Donatelli, Guo and Webster, 1991; Gorman, Donis, Kawaoka and Webster, 1990; Kawaoka, Krauss and Webster, 1989; Russell and Webster, 2005). This has occurred at least three times in the twentieth century, producing the pandemics of 1918, 1957 and 1968. All three of these pandemic strains were of avian origin but, perhaps involved additional hosts such as pigs. We are only beginning to address many of the questions that need to be answered in order to be prepared for and, we hope, to prevent the next pandemic. What are the sufficient and necessary conditions for an Influenza virus to cross from one host to another? How often does this happen? One type of avian Influenza virus, H5N1, has recently been isolated from human hosts and implicated in a considerable number of deaths (see Fig. 1). At the present time, no human to human transmission has been reported. In light of these facts, it has become an urgent issue to understand these types of host-virus interactions.

The second key feature of Influenza evolution is its very high mutation rate (close to 1 error per replication) (Drake, 1993; Drake and Holland, 1999; Nobusawa and Sato, 2006; Parvin, Moscona, Pan, Leider and Palese, 1986). Rapidly accruing point mutations are one of the causes of antigenic drift, i.e. the evolution of the surface proteins of the virion, hemagglutinin and neuraminidase. Is this drift random? Is it different in different hosts? Are there preferred directions in the evolution of the virus that can help us to understand its origin and predict its future? Every year Influenza comes back in a slightly different form from the previous year. This is one of the reasons that the vaccine has to be updated every couple of years. In temperate regions, the peak of the epidemic is in the winter months (January and February in the North Hemisphere and July–August in the Southern Hemisphere). This seasonal behavior contrasts with the constant background activity in Tropical Regions, (Alonso, Viboud, Simonsen, Hirano, Daufenbach and Miller, 2007; Nelson and Holmes, 2007; Viboud, Alonso and Simonsen, 2006; Wong, Yang, Chan, Leung, Chan, Guan, Lam, Hedley and Peiris, 2006). Despite the different seasonal behavior in tropical and non-tropical regions, annual infection rates and symptoms are similar. Why is epidemic Influenza seasonal? Is there an accurate method to predict next season’s primary strains?

The third feature of Influenza evolution is the reassortment of the viral chromosomes. The genome of the Influenza virus contains eight single stranded negative RNA segments coding for ten or eleven proteins. When two or more different Influenza viruses co-infect the same host cell, new virions are produced that can contain the RNA from a combination of segments from all the parental strains (see Fig. 2). This mode of evolution is related to antigenic shift and it has caused at least two of the pandemics in the twentieth century. For instance, in 1957, the Asian flu was a reassorted virus containing three segments from an avian strain (PB1, HA and NA) and the other five from the virus that was already circulating in the human population (H1N1).

In this review we will discuss some recent advances related to the three features of Influenza evolution, host variety, high mutation rates, and reassortments. In the last few years a large international effort has been developed to make thousands of viral sequences publicly available. These sequences have been isolated all around the world during the past hundred years. Using the information in these databases we will present some of the patterns of evolution of this virus. The evolution of Influenza is not completely random, i.e. there are some structures or patterns that reflect the biology of the virus, its interaction with different hosts and their immune systems.

## Influenza and Its Different Hosts

Influenza is an RNA- (antisense) virus with a genome fragmented into eight different segments. These segments contain 10 or 11 open reading frames. The 3 longest segments contain the genes coding for the polymerase complex (PB2, PB1 and PA). Two other segments code for the proteins in the envelope of the virus Hemagglutinin (HA) and Neuraminidase (NA). These two proteins play a crucial role in the interaction of the virus with the host cell and the host immune system. Two genes in the same segment code for the two proteins that form the capsid (M1 and M2). The other three or four proteins, are ribonucleoprotein (NP) and proteins (NS1, NS2 and PB1-F2) that are not incorporated in the viral particle but are important in the interaction with the host cell. PB1-F2 is a proapoptotic protein that is not present in all Influenza A viruses (Chen, Calvo, Malide, Gibbs, Schubert, Bacik, Basta, O’Neill, Schickli, Palese et al. 2001; Zell, Krumbholz, Eitner, Krieg, Halbhuber and Wutzler, 2007). It is encoded in an alternative reading frame in the same segment that is encoding PB1. Classic swine and human H1N1 viruses have chain termination or STOP codons mutations in the middle of the gene.

The nomenclature for the virus comes from the serotype classification based on the antibody response from the proteins on the surface of these viruses. There are 16 types of Hemagglutinins and 9 types of Neuraminidases. All of them can be found in birds but only H1, H2, H3 and N1 and N2 have been found in human epidemic Influenza. The prevalence of infection in certain avian populations, the wide variety of viral subtypes found, and a weaker immune response lead us to believe that the main reservoir of Influenza A is aquatic birds. Although in these birds transmission is through an oral-fecal route, in humans the virus spreads through droplets coming from the upper respiratory tract of an infected person. Occasionally an Influenza virus from one host jumps into a different host population. That happened in 1957 and in 1968 when several segments (PB1, HA and NA in 1957 and PB1 and HA in 1968) from avian viruses reassorted with the pre-existing human viruses. More controversial is the possibility of the direct jump of a complete Influenza virus to a new host. Taubenberger et al. have argued that the H1N1 flu causing the 1918 pandemic (Spanish flu) was an avian flu that entered the human population (Taubenberger, Reid, Lourens, Wang, Jin and Fanning, 2005). This possibility has been questioned by several authors (Antonovics, Hood and Baker, 2006; Gibbs and Gibbs, 2006; Tumpey, Basler, Aguilar, Zeng, Solorzano, Swayne, Cox, Katz, Taubenberger, Palese et al. 2005).

It is not clear what the necessary and sufficient conditions are that define the host specificity of one virus. One of these conditions is the receptor on the surface of the host cell, the sialic acid, which presents two versions alpha2–6 and alpha2–3, the former more common in humans and the latter in birds. Several positions have been mapped on the Hemagglutinin related to sialic acid specificity (Stevens, Blixt, Tumpey, Taubenberger, Paulson and Wilson, 2006).

## The Drift of Influenza

Recent bioinformatics research has illuminated some genomic features that distinguish avian and human Influenza A viruses. Viruses whose primary hosts are avian or human have different nucleotide compositions (Rabadan, Levine and Robins, 2006). This difference in nucleotide composition is sufficient to separate the thousands of sequenced human and avian viruses at almost 100% accuracy (See Fig. 3). The four sets of strains that fail to be classified by this method are H5N1 Hong Kong, H9N2 Hong Kong, the recent H5N1 bird flu, and the 1918 H1N1 virus. These are all known to have been avian viruses that recently had entered the human population and were not able to transmit from human to human, with the sole exception of the 1918 H1N1 virus. If we can understand why and how these viruses crossed over the avian to human line, then we will have a new tool to identify possible threats for new pandemics. Segment by segment analysis of nucleotide composition allows us to readily determine the reassortment of an avian segment into a human strain such as the PB1 gene on segment 2 in the 1957 and 1968 pandemics.

The human viruses have a higher percentage of Uracil and Adenine, whereas the avian viruses have a higher percentage of Guanine and Cytosine in their genomes (See Fig. 4) (Rabadan, Levine and Robins, 2006). One or more segments in each human strain were acquired by reassortment from a non-human virus, possibly avian. The nucleotide composition changes in the reassorted segments, probably due to a biased substitution rate (C->U and G->A) in human hosts relative to avian. Because of the availability of sequenced strains that span the last 90 years, we actually can observe the steady increase of U and A along with the decrease in C and G over time as the viral subtype evolves in its human host (Rabadan, Levine and Robins, 2006). A nice example is the H1N1 subtype that entered the human population in just prior 1918. The original 1918 strain, recently sequenced from lung tissue found in several victims of the Spanish flu in Alaska, has an avian nucleotide composition in the set of statistically resolvable segments, which include PB2, PB1, PA, and NP. As we follow the sequenced H1N1 strains for the next 90 years, the composition shifts until it reaches the present day composition, which is entirely human. Computing the rate of substitution from the early strains, the final steady state nucleotide composition for this strain is determined. The nucleotide composition of the present day strains are within the upper bound provided by this calculation (Rabadan, Levine and Robins, 2006).

The observed bias in the rates of fixation of nucleotides C and U in human versus avian Influenza A viruses has three different potential explanations. One possibility is natural selection. The cellular environment in humans favors more U’s and fewer C’s relative to avian for the Influenza virus. Perhaps this could be due to temperature differences which affect RNA structure. However, the evidence presented suggests that the changes are not due to positive selection because most of the mutations are found in third codon positions, consistent with neutral changes. Another possibility is that the RNA-RNA polymerase machinery includes different cellular components in human and avian cells, creating a relative mutation bias. The final possibility, which we find the most intriguing, is that humans have a native defense against RNA viruses that operates in a manner similar to the Apobec family of genes (Cullen, 2006; Sawyer, Emerman and Malik, 2004; Yu, Konig, Pillai, Chiles, Kearney, Palmer, Richman, Coffin and Landau, 2004). The Apobec3G gene is known to cause deamination of Cytosine which results in a Uracil during the retrotranscription of Lentiviruses. The Apobec gene family does not appear to have orthologs in avian species.

There have been some additional recent advances in the study of Influenza mutation presented in a series of works by Wu and Yan that are not discussed here (Wu and Yan, 2006a; Wu and Yan, 2006b). Also, the role of secondary structure has been studied in recent works, but is beyond the scope of this review (Wei, Du, Sun and Chou, 2006).

## Antigenic Shift and Reassortments

**Figure 2.**
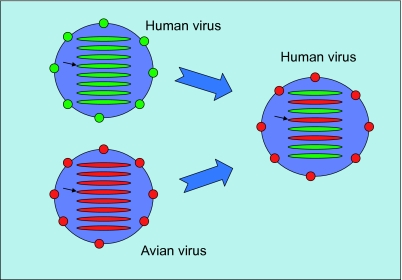
Reassortment: when two different virus co-infect the same host cell they produce a new virus with a combination of both parental strains. When a virus from a host reassorts with a virus from another host they can create a potential pandemic virus. In this example, a human virus reassorted with an avian virus taking three of its segments. In particular, the segment coding for HA (indicated by a black arrow) in the resulting virus is of avian origin. A similar process happened in 1968 (the Asian flu).

**Figure 3.**
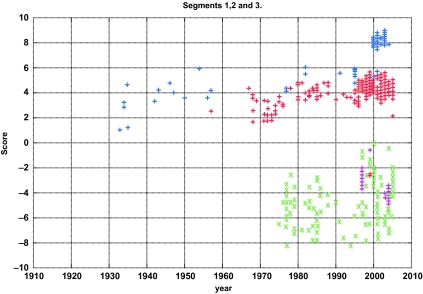
The log-odds score of Human and Avian Influenza A virus’ nucleotide composition from the coding sequences of the polymerase genes versus year. Blue asterisks are Human H1N1 strains, purple squares are H5N1 found in humans, and red pluses are the remaining human strains available from the NCBI database. Green crosses are all the avian strains available in the NCBI database at the time of analysis (Rabadan, Levine and Robins, 2006).

**Figure 4.**
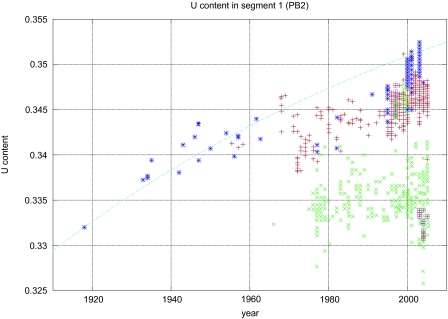
The U content evolution of segment 1, PB2. Blue asterisks are Human H1N1 strains, purple squares are H5N1 found in humans, and red pluses are the remaining human strains available from the NCBI database. Green crosses are all the avian strains available in the NCBI database at the time of analysis. The blue dashed line is the predicted evolutionary curve for U content change computed using the U, C block diagonal component of the substitution matrices. This matrix was derived from the nucleotide content of the 1918 H1N1 and 1933 H1N1 Wilson-Smith strains (Rabadan, Levine and Robins, 2006).

When two different Influenza A viruses co-infect the same host cell, new virions are released that contain segments from both parental strains (see Fig. 2). This is the main way Influenza viruses exchange genetic material, a process known as reassortment (Holmes, Ghedin, Miller, Taylor, Bao, St George, Grenfell, Salzberg, Fraser, Lipman et al. 2005; Lindstrom, Cox and Klimov, 2004; Schweiger, Bruns and Meixenberger, 2006). At least two of the major Influenza pandemics of the twentieth century, H2N2 in 1957 and H3N2 in 1968, resulted from reassortments between viruses from two different hosts, avian and human. Within human viruses, reassortments have been related to some of the failures of Influenza vaccine prediction, as in 2003. How often do these reassortments occur? Are all possible reassortments equally likely or are there preferred patterns? If we combine two viruses we expect the reassortants to follow a binomial distribution, i.e. roughly four segments from each parental strain.

To answer these questions, M. Lubeck, P. Palese and J. Schulman analyzed 40 reassortant viruses derived from A/PR/8/34 (H1N1) and A/HK/8/68 (H3N2) in the laboratory (Lubeck, Palese and Schulman, 1979). They found strong correlations among segments 1, 2 and 3, 1 and 5, and 3 and 8. Are these results universal? Do they only apply to a particular pair of Influenza strains? Can they be found in vivo during local epidemics? Another issue is that the patterns of reassortment observed *in vitro*, in cell culture, are not subject to immunoselection or other forces that may act *in vivo* in human hosts. To understand the patterns of reassortment of viral populations one has to provide quantitative answers.

The most traditional way of detecting reassort ments is by constructing phylogenetic trees for the whole genome, as well as for each viral segment, and looking for strains that have segments on different branches of their respective trees (Holmes, Ghedin, Miller, Taylor, Bao, St George, Grenfell, Salzberg, Fraser, Lipman et al. 2005; Lindstrom, Cox and Klimov, 2004; Lindstrom, Hiromoto, Nerome, Omoe, Sugita, Yamazaki, Takahashi and Nerome, 1998; Nelson, Simonsen, Viboud, Miller, Taylor, George, Griesemer, Ghedin, Sengamalay, Spiro et al. 2006; Schweiger, Bruns and Meixenberger, 2006). There are several limitations to this approach: the structure of a phylogenetic tree depends on the method used to construct it and mutational biases can make accurate phylogenetic analysis very challenging.

**Figure 5.**
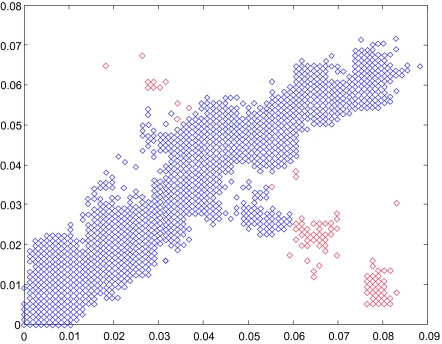
Hamming distance in third codon position between human H3N2 Influenza strains in the New York state (208 sequences from 2000–2003) in segment 1 versus segment 3. Mutations accumulate at similar rates in different segments. That makes that most of the pairs are distributed along the diagonal. When reassortments occur this pattern is violated and the exchange of segments produce pairs of viruses where the distances in different segments are not proportional to each other. Reassortments appear as points outside of the diagonal. We can then proceed to analyze the sequences that are the origin of points. In red are the pairs of sequences that contain A/New York/11/ 2003(H3N2) (Rabadan, Levine and Robins, 2006).

If our only goal is detecting likely reassortments, there is no need to go through the intermediate step of tree inference. For instance, we can compare genetic distances in pairs of viruses in different segments (Rabadan, Levine and Kraznitz). As viruses replicate over time their sequences change and knowing the evolutionary rates in every segment one can estimate how likely it is that a particular set of distances happens by random chance. For example, let us take two segments, segment 1 (coding for one of the polymerases, PB2) vs segment 3 for human H3N2 Influenza strains in New York State (208 sequences from 2000–2003) (See Fig. 5). To avoid selection pressures we only consider third codon positions. We take every pair of virus and we compute the changes between these two viruses in segment 1 and in segment 3. If there are no reassortments the distances should form a straight line and deviations from this line indicate a possible reassortment. In Figure 5, we can see that while although most of the points are along the line with slope one (45 degrees), there are many points lying significantly off the diagonal. Most of the points that reside off the diagonal come from pairs containing a single strain, A/New York/11/2003(H3N2) (in Fig. 5 the pairs of sequences that contain A/New York/11/2003(H3N2) are marked in red). This is a clear indication that A/New York/11/2003(H3N2) is a reassortment that involved segment 1 or segment 3. We can construct statistical tests that measure how probable is that a particular pair deviates from the diagonal and using these tests we can systematically extract all the cases that can be identified as reassortants. With the list of reassortments, we can estimate how likely it is that the reassortment process is random (in this case binomial), what are the correlations between different segments and what are the reassortment rates.

There are several possible explanations for the fact that the reassortment process is not random. The first possible explanation is that the interactions between the different proteins of the virus demand compensatory mutations. For instance, we know that the three polymerases form a complex of proteins and that they work together. Mutations in one amino acid in the interaction domain in one of these polymerases can be compensated by mutations in other polymerase. Another possible explanation comes from the fact that the interaction with the host cell requires host and tissue specificity. To infect a particular host or cell we need a particular combination of proteins that is well adapted for the growth of the virus in this cell. Another possible explanation comes from the process of packaging the eight different RNAs in the virion. The mechanism controlling how this could happen is not clear although two different hypotheses have been put forward: random packaging and specific signals (Bancroft and Parslow, 2002; Fujii, Fujii, Noda, Muramoto, Watanabe, Takada, Goto, Horimoto and Kawaoka, 2005; Fujii, Goto, Watanabe, Yoshida and Kawaoka, 2003; Gog, Afonso Edos, Dalton, Leclercq, Tiley, Elton, von Kirchbach, Naffakh, Escriou and Digard, 2007; Liang, Hong and Parslow, 2005; Muramoto, Takada, Fujii, Noda, Iwatsuki-Horimoto, Watanabe, Horimoto, Kida and Kawaoka, 2006; Noda, Sagara, Yen, Takada, Kida, Cheng and Kawaoka, 2006; Odagiri and Tashiro, 1997; Zheng, Palese and Garcia-Sastre, 1996).

## Conclusions, Open Problems and Future Directions

This review discusses high mutation rates and reassortments as the main mechanisms of evolution of Influenza. Are these the only two modes of evolution of this virus? Two cases have been reported of non-homologous recombination in avian Influenza viruses, one in 2002 in Chile and the other in 2004 in British Columbia, Canada (Pasick, Handel, Robinson, Copps, Ridd, Hills, Kehler, Cottam-Birt, Neufeld, Berhane et al. 2005; Suarez, Senne, Banks, Brown, Essen, Lee, Manvell, Mathieu-Benson, Moreno, Pedersen et al. 2004). In both cases, a low pathogenic avian Influenza virus (LPAI) mutated into a more virulent form (high pathogenic or HPAI). When these viruses were sequenced the only difference that was found was an extra insertion of a few amino acids in the cleavage site of Hemagglutinin. The extra sequence was incorporated from other segments (MP and NP). Apart from other reported cases in the laboratory it is clear that non-homologous recombination is not a very common phenomenon. More controversial is the possibility of homologous recombination (Chare, Gould and Holmes, 2003). Homologous recombination is an important mode of evolution in retroviruses (e.g. HIV), however for Influenza this has not been found in the laboratory.

The increasing number of publicly available isolates allows viral spread to be tracked throughout the world in humans, birds and other hosts (Chen, Smith, Li, Wang, Fan, Rayner, Vijaykrishna, Zhang, Zhang, Guo et al. 2006; Gaidet, Dodman, Caron, Balanca, Desvaux, Goutard, Cattoli, Lamarque, Hagemeijer and Monicat 2007; Ghedin, Sengamalay, Shumway, Zaborsky, Feldblyum, Subbu, Spiro, Sitz, Koo, Bolotov et al. 2005; Krauss, Walker, Pryor, Niles, Chenghong, Hinshaw and Webster, 2004; Munster, Baas, Lexmond, Waldenstrom, Wallensten, Fransson, Rimmelzwaan, Beyer, Schutten, Olsen et al. 2007; Munster, Veen, Olsen, Vogel, Osterhaus and Fouchier, 2006; Obenauer, Denson, Mehta, Su, Mukatira, Finkelstein, Xu, Wang, Ma, Fan et al. 2006; Olsen, Munster, Wallensten, Waldenstrom, Osterhaus and Fouchier, 2006; Wallensten, Munster, Latorre-Margalef, Brytting, Elmberg, Fouchier, Fransson, Haemig, Karlsson, Lundkvist et al. 2007). For the first time, epidemiological models can be tested accurately. We are not yet able to address many of the important questions about Influenza evolution: why the epidemic is seasonal, why it has a bottleneck structure (Many related human viruses are found every year but only a few of them are isolated the next year. This phenomenon makes the tree structure of human Influenza viruses to be more like a cactus than a tree.), and how the tropics are involved in Influenza evolution. Understanding these factors is key to fighting the epidemics of this virus and to developing more accurate vaccine prediction tools.

We have seen how the two main modes of evolution of Influenza present non-random patterns. Influenza viruses replicating in humans become more U rich in their genome. That allows us to understand a particular direction in the evolution of human Influenza, to understand the past and to predict the future of these viruses. Reassortments are not random processes, not *in vivo* or *in vitro*. The sequence space of different Influenza viruses is enormous and we are only touching the tip of the iceberg. Thanks to the worldwide sequencing effort and the amount of information that is publicly available, we can start answering some of the questions about this virus, its host and its evolution.

## Figures and Tables

**Figure 1.**
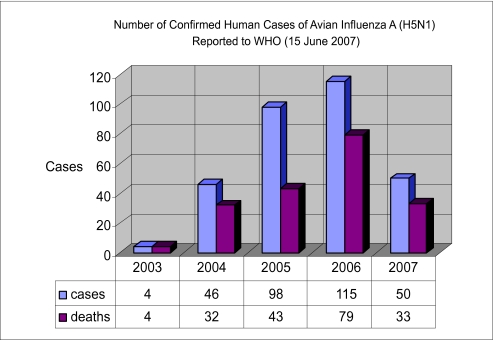
Human H5N1 cases reported by the World Health Organization (WHO) from 2003 till the 15th of June of 2007.

## References

[b1-ebo-03-299] Cumulative Number of Confirmed Human Cases of Avian Influenza A/(H5N1) Reported to WHO, World Health Organization.

[b2-ebo-03-299] Alonso WJ, Viboud C, Simonsen L, Hirano EW, Daufenbach LZ, Miller MA (2007). Seasonality of influenza in Brazil: a traveling wave from the Amazon to the subtropics. Am. J. Epidemiol.

[b3-ebo-03-299] Antonovics J Hood ME Baker CH 2006 Molecular virology: was the 1918 flu avian in origin? Nature 440 E9discussion E9–10. 16641950 10.1038/nature04824

[b4-ebo-03-299] Bancroft CT, Parslow TG (2002). Evidence for segment-nonspecific packaging of the influenza a virus genome. J. Virol.

[b5-ebo-03-299] Chare ER, Gould EA, Holmes EC (2003). Phylogenetic analysis reveals a low rate of homologous recombination in negative-sense RNA viruses. J. Gen Virol.

[b6-ebo-03-299] Chen H, Smith GJ, Li KS, Wang J, Fan XH, Rayner JM, Vijaykrishna D, Zhang JX, Zhang LJ, Guo CT (2006). Establishment of multiple sublineages of H5N1 influenza virus in Asia: implications for pandemic control. Proc. Natl. Acad. Sci. U.S.A.

[b7-ebo-03-299] Chen W, Calvo PA, Malide D, Gibbs J, Schubert U, Bacik I, Basta S, O’Neill R, Schickli J, Palese P (2001). A novel Influenza A virus mitochondrial protein that induces cell death. Nat. Med.

[b8-ebo-03-299] Cullen BR (2006). Role and mechanism of action of the APOBEC3 family of antiretroviral resistance factors. J. Virol.

[b9-ebo-03-299] Drake JW (1993). Rates of spontaneous mutation among RNA viruses. Proc. Natl. Acad. Sci. U.S.A.

[b10-ebo-03-299] Drake JW, Holland JJ (1999). Mutation rates among RNA viruses. Proc. Natl. Acad. Sci. U.S.A.

[b11-ebo-03-299] Fitch WM (1996). The variety of human virus evolution. Mol. Phylogenet. Evol.

[b12-ebo-03-299] Fujii K, Fujii Y, Noda T, Muramoto Y, Watanabe T, Takada A, Goto H, Horimoto T, Kawaoka Y (2005). Importance of both the coding and the segment-specific noncoding regions of the influenza A virus NS segment for its efficient incorporation into virions. J. Virol.

[b13-ebo-03-299] Fujii Y, Goto H, Watanabe T, Yoshida T, Kawaoka Y (2003). Selective incorporation of influenza virus RNA segments into virions. Proc. Natl. Acad. Sci. U.S.A.

[b14-ebo-03-299] Gaidet N, Dodman T, Caron A, Balanca G, Desvaux S, Goutard F, Cattoli G, Lamarque F, Hagemeijer W, Monicat F (2007). Avian influenza viruses in water birds, Africa. Emerg. Infect. Dis.

[b15-ebo-03-299] Gammelin M, Altmuller A, Reinhardt U, Mandler J, Harley VR, Hudson PJ, Fitch WM, Scholtissek C (1990). Phylogenetic analysis of nucleoproteins suggests that human influenza A viruses emerged from a 19th-century avian ancestor. Mol. Biol. Evol.

[b16-ebo-03-299] Ghedin E, Sengamalay NA, Shumway M, Zaborsky J, Feldblyum T, Subbu V, Spiro DJ, Sitz J, Koo H, Bolotov P (2005). Large-scale sequencing of human influenza reveals the dynamic nature of viral genome evolution. Nature.

[b17-ebo-03-299] Gibbs MJ Gibbs AJ 2006 Molecular virology: was the 1918 pandemic caused by a bird flu? Nature 440 E8discussion E9–10. 16641948 10.1038/nature04823

[b18-ebo-03-299] Gog JR, Afonso Edos S, Dalton RM, Leclercq I, Tiley L, Elton D, von Kirchbach JC, Naffakh N, Escriou N, Digard P (2007). Codon conservation in the influenza A virus genome defines RNA packaging signals. Nucleic Acids Res.

[b19-ebo-03-299] Gorman OT, Bean WJ, Kawaoka Y, Donatelli I, Guo YJ, Webster RG (1991). Evolution of influenza A virus nucleoprotein genes: implications for the origins of H1N1 human and classical swine viruses. J. Virol.

[b20-ebo-03-299] Gorman OT, Donis RO, Kawaoka Y, Webster RG (1990). Evolution of influenza A virus PB2 genes: implications for evolution of the ribonucleoprotein complex and origin of human influenza A virus. J. Virol.

[b21-ebo-03-299] Holmes EC, Ghein E, Miller N, Taylor J, Bao Y, St George K, Grenfell BT, Salzberg SL, Fraser CM, Lipman DJ (2005). Whole-genome analysis of human influenza A virus reveals multiple persistent lineages and reassortment among recent H3N2 viruses. PLoS Biol.

[b22-ebo-03-299] Kamps BS Hoffmann C Preiser W 2006Influenza Report.

[b23-ebo-03-299] Kawaoka Y, Krauss S, Webster RG (1989). Avian-to-human transmission of the PB1 gene of influenza A viruses in the 1957 and 1968 pandemics. J. Virol.

[b24-ebo-03-299] Krauss S, Walker D, Pryor SP, Niles L, Chenghong L, Hinshaw VS, Webster RG (2004). Influenza A viruses of migrating wild aquatic birds in North America. Vector Borne Zoonotic Dis.

[b25-ebo-03-299] Liang Y, Hong Y, Parslow TG (2005). cis-Acting packaging signals in the influenza virus PB1, PB2, and PA genomic RNA segments. J. Virol.

[b26-ebo-03-299] Lindstrom SE, Cox NJ, Klimov A (2004). Genetic analysis of human H2N2 and early H3N2 influenza viruses, 1957–1972: evidence for genetic divergence and multiple reassortment events. Virology.

[b27-ebo-03-299] Lindstrom SE, Hiromoto Y, Nerome R, Omoe K, Sugita S, Yamazaki Y, Takahashi T, Nerome K (1998). Phylogenetic analysis of the entire genome of influenza A (H3N2) viruses from Japan: evidence for genetic reassortment of the six internal genes. J. Virol.

[b28-ebo-03-299] Lipatov AS, Govorkova EA, Webby RJ, Ozaki H, Peiris M, Guan Y, Poon L, Webster RG (2004). Influenza: emergence and control. J. Virol.

[b29-ebo-03-299] Lubeck MD, Palese P, Schulman JL (1979). Nonrandom association of parental genes in influenza A virus recombinants. Virology.

[b30-ebo-03-299] Munster VJ, Baas C, Lexmond P, Waldenstrom J, Wallensten A, Fransson T, Rimmelzwaan GF, Beyer WE, Schutten M, Olsen B (2007). Spatial, temporal, and species variation in prevalence of influenza A viruses in wild migratory birds. PLoS Pathog.

[b31-ebo-03-299] Munster VJ, Veen J, Olsen B, Vogel R, Osterhaus AD, Fouchier RA (2006). Towards improved influenza A virus surveillance in migrating birds. Vaccine.

[b32-ebo-03-299] Muramoto Y, Takada A, Fujii K, Noda T, Iwatsuki-Horimoto K, Watanabe S, Horimoto T, Kida H, Kawaoka Y (2006). Hierarchy among viral RNA (vRNA) segments in their role in vRNA incorporation into influenza A virions. J. Virol.

[b33-ebo-03-299] Nelson MI, Holmes EC (2007). The evolution of epidemic influenza. Nat. Rev. Genet.

[b34-ebo-03-299] Nelson MI, Simonsen L, Viboud C, Miller MA, Taylor J, George KS, Griesemer SB, Ghedin E, Sengamalay NA, Spiro DJ (2006). Stochastic processes are key determinants of short-term evolution in influenza a virus. PLoS Pathog.

[b35-ebo-03-299] Nobusawa E, Sato K (2006). Comparison of the mutation rates of human influenza A and B viruses. J. Virol.

[b36-ebo-03-299] Noda T, Sagara H, Yen A, Takada A, Kida H, Cheng RH, Kawaoka Y (2006). Architecture of ribonucleoprotein complexes in influenza A virus particles. Nature.

[b37-ebo-03-299] Obenauer JC, Denson J, Mehta PK, Su X, Mukatira S, Finkelstein DB, Xu X, Wang J, Ma J, Fan Y (2006). Large-scale sequence analysis of avian influenza isolates. Science.

[b38-ebo-03-299] Odagiri T, Tashiro M (1997). Segment-specific noncoding sequences of the influenza virus genome RNA are involved in the specific competition between defective interfering RNA and its progenitor RNA segment at the virion assembly step. J. Virol.

[b39-ebo-03-299] Olsen B, Munster VJ, Wallensten A, Waldenstrom J, Osterhaus AD, Fouchier RA (2006). Global patterns of influenza a virus in wild birds. Science.

[b40-ebo-03-299] Parvin JD, Moscona A, Pan WT, Leider JM, Palese P (1986). Measurement of the mutation rates of animal viruses: influenza A virus and poliovirus type 1. J. Virol.

[b41-ebo-03-299] Pasick J, Handel K, Robinson J, Copps J, Ridd D, Hills K, Kehler H, Cottam-Birt C, Neufeld J, Berhane Y (2005). Intersegmental recombination between the haemagglutinin and matrix genes was responsible for the emergence of a highly pathogenic H7N3 avian influenza virus in British Columbia. J. Gen. Virol.

[b42-ebo-03-299] Rabadan R Levine AJ Kraznitz MNon-Random Reassortment in Human Influenza A Viruse. 10.1111/j.1750-2659.2007.00030.x PMC4634327 19453489

[b43-ebo-03-299] Rabadan R, Levine AJ, Robins H (2006). Comparison of avian and human influenza A viruses reveals a mutational bias on the viral genomes. J. Virol.

[b44-ebo-03-299] Russell CJ, Webster RG (2005). The genesis of a pandemic influenza virus. Cell.

[b45-ebo-03-299] Sawyer SL, Emerman M, Malik HS (2004). Ancient adaptive evolution of the primate antiviral DNA-editing enzyme APOBEC3G. PLoS Biol.

[b46-ebo-03-299] Schweiger B, Bruns L, Meixenberger K (2006). Reassortment between human A(H3N2) viruses is an important evolutionary mechanism. Vaccine.

[b47-ebo-03-299] Stevens J, Blixt O, Tumpey TM, Taubenberger JK, Paulson JC, Wilson IA (2006). Structure and receptor specificity of the hemagglutinin from an H5N1 influenza virus. Science.

[b48-ebo-03-299] Suarez DL, Senne DA, Banks J, Brown IH, Essen SC, Lee CW, Manvell RJ, Mathieu-Benson C, Moreno V, Pedersen JC (2004). Recombination resulting in virulence shift in avian influenza outbreak, Chile. Emerg. Infect Dis.

[b49-ebo-03-299] Taubenberger JK, Reid AH, Lourens RM, Wang R, Jin G, Fanning TG (2005). Characterization of the 1918 influenza virus polymerase genes. Nature.

[b50-ebo-03-299] Tumpey TM, Basler CF, Aguilar PV, Zeng H, Solorzano A, Swayne DE, Cox NJ, Katz JM, Taubenberger JK, Palese P (2005). Characterization of the reconstructed 1918 Spanish influenza pandemic virus. Science.

[b51-ebo-03-299] Viboud C, Alonso WJ, Simonsen L (2006). Influenza in tropical regions. PLoS Med.

[b52-ebo-03-299] Wallensten A, Munster VJ, Latorre-Margalef N, Brytting M, Elmberg J, Fouchier RA, Fransson T, Haemig PD, Karlsson M, Lundkvist A (2007). Surveillance of influenza A virus in migratory waterfowl in northern Europe. Emerg. Infect Dis.

[b53-ebo-03-299] Webster RG, Bean WJ, Gorman OT, Chambers TM, Kawaoka Y (1992). Evolution and ecology of influenza A viruses. Microbiol Rev.

[b54-ebo-03-299] Wei DQ, Du QS, Sun H, Chou KC (2006). Insights from modeling the 3D structure of H5N1 influenza virus neuraminidase and its binding interactions with ligands. Biochem. Biophys. Res. Commun.

[b55-ebo-03-299] Wong CM, Yang L, Chan KP, Leung GM, Chan KH, Guan Y, Lam TH, Hedley AJ, Peiris JS (2006). Influenza-associated hospitalization in a subtropical city. PLoS Med.

[b56-ebo-03-299] Wu G, Yan S (2006a). Fate of Influenza A virus proteins. Protein Pept. Lett.

[b57-ebo-03-299] Wu G, Yan S (2006b). Prediction of mutations in H5N1 hemagglutinins from influenza A virus. Protein Pept. Lett.

[b58-ebo-03-299] Yu Q, Konig R, Pillai S, Chiles K, Kearney M, Palmer S, Richman D, Coffin JM, Landau NR (2004). Single-strand specificity of APOBEC3G accounts for minus-strand deamination of the HIV genome. Nat. Struct. Mol. Biol.

[b59-ebo-03-299] Zell R, Krumbholz A, Eitner A, Krieg R, Halbhuber KJ, Wutzler P (2007). Prevalence of PB1-F2 of influenza A viruses. J. Gen. Virol.

[b60-ebo-03-299] Zheng H, Palese P, Garcia-Sastre A (1996). Nonconserved nucleotides at the 3′ and 5′ ends of an influenza A virus RNA play an important role in viral RNA replication. Virology.

